# What Enables True “Respite”? Mediation Pathways of Caregiver Well-Being and Supply-Demand Matching in Respite Care

**DOI:** 10.1093/geroni/igaf122.854

**Published:** 2025-12-31

**Authors:** Chen Chen, Xiuting Cai, Guoyong Ma, Hong Mi

**Affiliations:** School of Public Administration, Zhejiang University, Hangzhou, Zhejiang, China (People’s Republic); College of Economics and Management, Northeast Forestry University, Harbin City, Heilongjiang, China (People’s Republic); Northeast Forestry University, Harbin, Heilongjiang, China (People’s Republic); Zhejiang University, Hangzhou, Zhejiang, China (People’s Republic)

## Abstract

The effectiveness of respite care, as an external support mechanism within family elderly care systems, not only directly impacts the multidimensional well-being of older individuals and their family caregivers but also bears significant relevance to regional social security and the long-term stability of population development. By deconstructing the core connotation of “respite”—encompassing the dimensions of “service utilization intensity” and “respite capacity”—this study empirically verifies the significant enhancement of caregiver well-being through respite effects, while controlling for heterogeneity in prior research conclusions. Notably, improvements in caregivers’ well-being can synchronously optimize elderly care quality through intergenerational interaction mechanisms, thereby establishing a bidirectional virtuous cycle. Through deconstructing caregiver well-being into three dimensions—positive affect, negative emotions, and abusive tendencies—path analysis reveals that caregivers’ respite capacity exerts significant effects across all three well-being indicators, with systematic variations in directional impact and magnitude. Besides, variables such as caregivers’ health status, familial support pressures, intergenerational relationship quality, economic burden levels, and older adults’ dependence on daily activities significantly influence well-being dimensions. Mediation effect analyses further demonstrate that intrinsic motivation mechanisms partially mediate the pathways through which familial support and parental support affect positive affect, while resource constraint mechanisms significantly mediate the transmission of economic pressures and daily activity dependence to negative emotions and abusive tendencies. Crucially, structural differences exist between the pathways of respite service utilization intensity and respite capacity: the former only significantly enhances positive affect, whereas its effects on mitigating negative emotions and abusive tendencies lack statistical significance, indicating functional limitations in current service models.

